# Epidemiology and associated factors of childhood chalazion in China: a seven-year, hospital-based, multicenter, cross-sectional study

**DOI:** 10.1016/j.jped.2026.101556

**Published:** 2026-05-29

**Authors:** Chang Liu, Xinyu Wang, Guoshuang Feng, Chunni Zhang, Li Li, Jingjing Jiang

**Affiliations:** a National Center for Children’s Health, Capital Medical University, Beijing Children’s Hospital, Department of Ophthalmology, Beijing, China; b Ministry of Education, Key Laboratory of Major Diseases in Children, Beijing, China; c National Center for Children's Health, Capital Medical University, Beiing Children's Hospital, Big Data Center, Beijing, China; d Peking University, Department of Sociology, Beijing, China; e Yangzhou Maternal and Child Health Care Hospital Affiliated to Yangzhou University, Yangzhou Women and Children’s Hospital, Yangzhou, Jiangsu, China

**Keywords:** Chalazion, Multicenter, Associated factors, Surgery

## Abstract

**Objective:**

To describe the epidemiology of childhood chalazion surgery under general anesthesia in China and identify factors associated with it.

**Method:**

The authors conducted a hospital-based, multicenter, cross-sectional study using retrospective inpatient data from the Futang Updating Medical Records System from 2016 to 2022. The study included 117,009 children who underwent ophthalmic surgery under general anesthesia at 22 tertiary hospitals. Age, sex, surgical season, and geographic region were analyzed.

**Results:**

Of 117,009 pediatric ophthalmic procedures performed under general anesthesia, 33,489 (28.62 %) were chalazion excisions. Annual proportions remained stable at 25-33%. Most children were 1 to 3 years old, with a mean age at surgery of 3.30 years. After adjustment, female residents in North China, and surgery in autumn or winter were each associated with higher odds of chalazion surgery under general anesthesia than their respective reference groups.

**Conclusions:**

Childhood chalazion surgery under general anesthesia accounted for a substantial share of pediatric ophthalmic surgical volume in this multicenter national cohort. The highest surgical burden was observed among toddlers, girls, children treated in North China, and those undergoing surgery in autumn and winter.

## Introduction

Chalazion, a term derived from the Greek word for “hailstone” or “small lump” [1], is characterized as a subacute, lipogranulomatous inflammation resulting from the obstruction of sebaceous glands in the eyelid, specifically the meibomian or Zeiss glands. This obstruction leads to the retention of secretions, which manifests as painless nodules on either the inner eyelid or the skin surface [2].

The pathogenesis of chalazion is frequently associated with ocular surface disorders, including chronic blepharitis, meibomian gland dysfunction (MGD), demodex infestation, rosacea, and VA deficiency [[3], [4], [5], [6], [7], [8]]. Chalazion can occur at any age and is often a self-limiting condition; however, in cases where these conservative approaches prove ineffective, surgical intervention may be warranted [9].

A retrospective study conducted in India, which analyzed data from 1,982,058 patients who underwent eye examinations in the multi-tier hospital network from 2010 to 2019, reported a prevalence rate of 0.6% (11,270 individuals). The findings indicated a higher incidence in children (1.0%; 2,656 out of 280,034) compared to adults (0.5%; 8,614 out of 1,702,024) [10]. Evans et al. reported a peak incidence of pediatric cases for surgical excision under general anesthesia among children aged 0-5 years, with a particular emphasis on those aged 2-5 years, in the United States [11]. In a retrospective study in China, 74.2% of children who underwent chalazion excision under general anesthesia were younger than 3 years [12]. Together, these data suggest that chalazion is particularly relevant in early childhood and that surgically managed cases are concentrated in younger children.

Management of pediatric chalazion is more challenging than in adults because parents frequently request surgery for cosmetic improvement, and early-onset disease may also impair children’s psychological development.

Due to the limited available information regarding the epidemiology of childhood chalazion and its associated factors, this multicenter retrospective study utilizes data from a national collaborative pediatric medical platform to examine the epidemiological characteristics of childhood chalazion requiring surgical intervention under anesthesia across various regions in China.

## Methods

### Study design and participants

**Figure 1:**
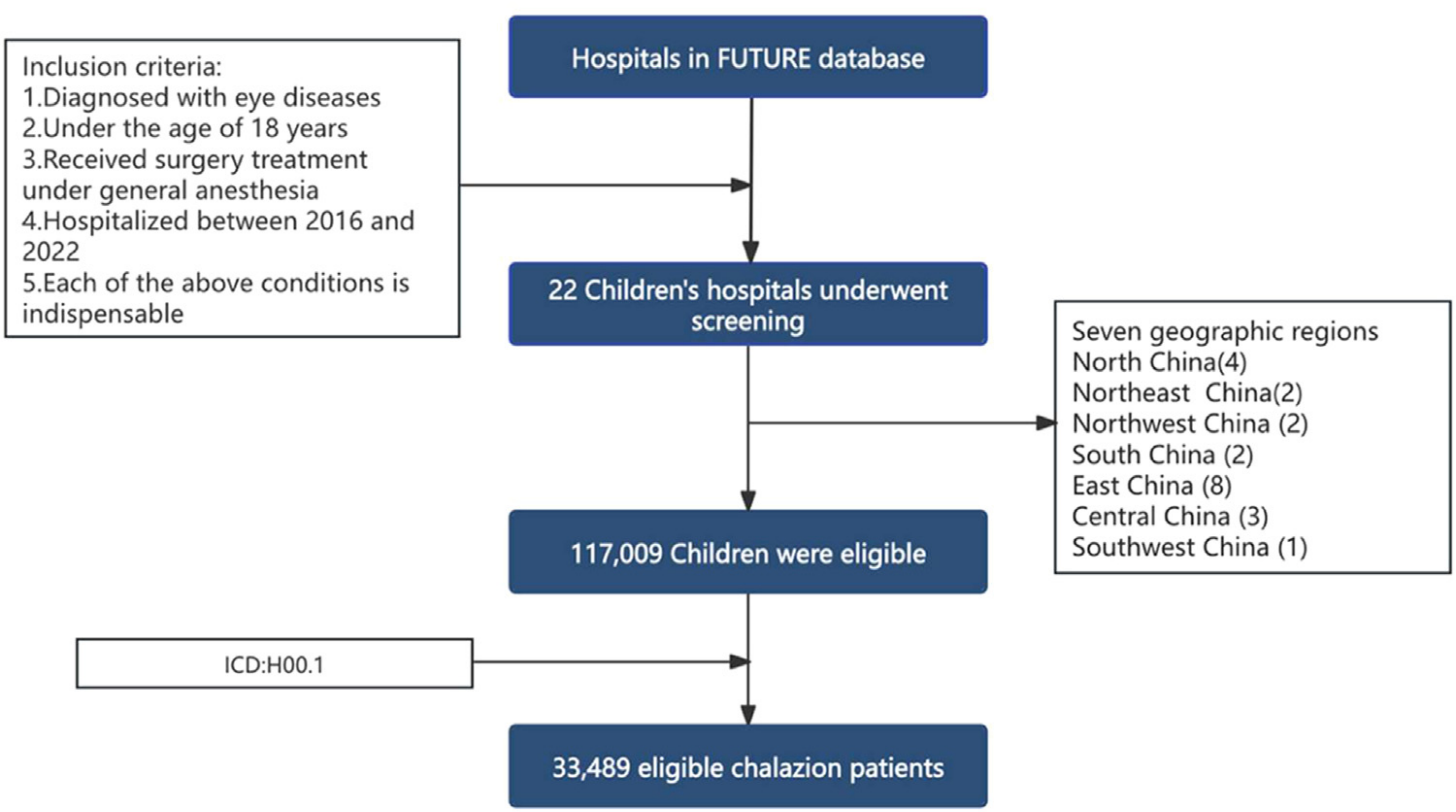
Flowchart utilized for selecting participants of chalazion.

This hospital-based cross-sectional study employed data from the Futang Updating Medical Records (FUTURE) system, which is managed by the Futang Research Center of Pediatric Development (FRCPD). The FRCPD is a non-profit organization supervised by the Ministry of Civil Affairs and is affiliated with the National Children's Medical Center. It comprises 47 provincial and municipal medical institutions, thereby establishing a national pediatric health network [13]. The authors conducted a retrospective review of the medical records. The inclusion criteria for participants in this study were as follows: (1) diagnosed with eye diseases; (2) under the age of 18 years; (3) received surgical treatment while under general anesthesia; (4) were hospitalized during the period from January 1, 2016, to December 31, 2022. Chalazion participants were required to meet criteria (1)-(4) above, and (5) cases were identified that underwent chalazion excision, utilizing the ICD-10 code H00.1 (chalazion) extracted from the medical record front sheets. To ensure data integrity and the comparability of the control group. Exclusion criteria for all participants included: (1) diagnosed with eye diseases but were exclusively treated in outpatient settings; (2) hospitalized but did not undergo surgical interventions. The data extracted for analysis included age, gender, date of surgery, and hospital location. Figure 1 illustrates the flowchart utilized for participant selection. This study was approved by the Medical Ethics Committee of Beijing Children’s Hospital, Capital Medical University (No. 2025-202-D). The requirement for informed consent was waived because of the retrospective nature of the study.

### Grouping by age, season, and geographic region

Age was divided into five subgroups, which include infants (under 1 year), toddlers (1 to 3 years), preschoolers (4 to 6 years), school-age children (7 to 12 years), and adolescents (13 to 18 years).

The season has been categorized into four subgroups based on astronomical and climatic parameters: Spring (March to May), Summer (June to August), Autumn (September to November), and Winter (December to February).

In mainland China, a commonly used 7-region geographic classification is widely applied in medical, meteorological, and national statistical research, specifically East China, South China, Central China, North China, Southwest China, Northwest China, and Northeast China. The geographic division between North and South China is demarcated by the Qinling Mountains-Huaihe River Line, primarily based on climatic criteria (the 800mm annual precipitation isohyet and 0°C January isotherm), natural landscapes, and agricultural practices [14]. Mapbox Studio version 9.6.0 was employed for Geographic Information System (GIS) mapping to illustrate the national distribution of cases. The cases were geocoded utilizing the latitude and longitude coordinates of the treating hospitals. An analysis of the distributions was conducted on the North-South divide.

### Statistical analysis

Continuous variables are expressed as mean ± standard deviation (SD), while categorical variables are represented as frequencies and percentages. The sex ratio was determined using the formula (number of males/number of females) × 100. The chi-square test was employed to assess differences across subgroups categorized by age, gender, season, and region. For post-hoc pairwise comparisons, the Bonferroni correction was applied to account for multiple testing, with a significance threshold set at *p* < α/n. To control for confounding, the authors built a multivariable logistic regression. A multivariate binary logistic regression analysis was performed to investigate the associations between chalazion surgery and the variables of gender, age group, season, and geographic region (North vs. South), with results reported as adjusted odds ratios (aOR) and 95% confidence intervals (CI). *p* < 0.05 is considered statistically significant.

## Results

**Figure 2:**
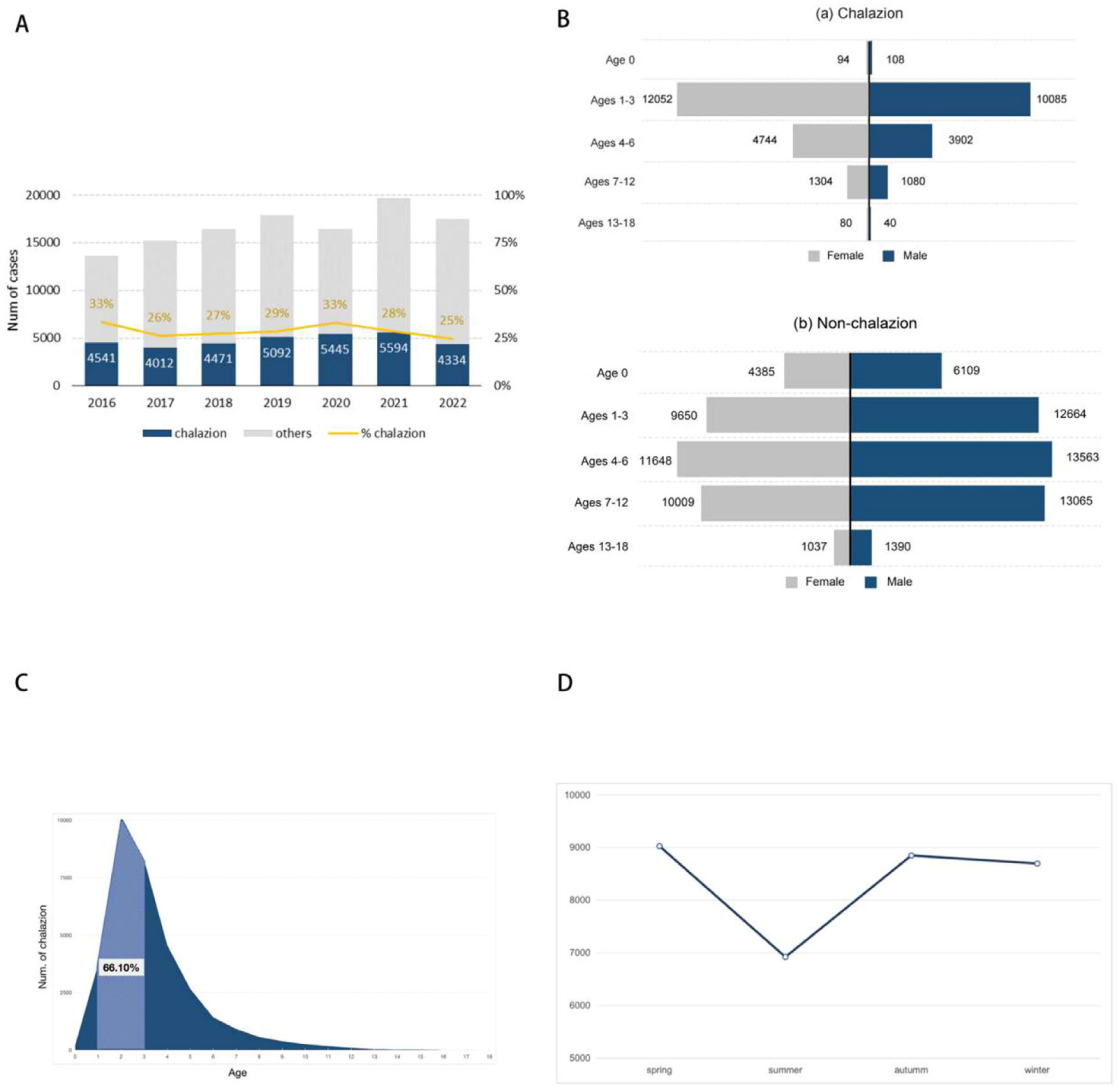
(A)The chalazion and non-chalazion cases and the proportion of chalazion surgeries among all ophthalmic surgeries under general anesthesia. (B) Gender distribution of children with chalazion and non-chalazion under general anesthesia. (C) Age distribution of children with chalazion surgery under general anesthesia. (D) The distribution of chalazion under general anesthesia by season.

Between 2016 and 2022, the FUTURE database documented a total of 117,009 pediatric patients who underwent ophthalmic surgery under general anesthesia across 22 participating tertiary hospitals throughout the country. Among these, 33,489 children underwent chalazion excision, which accounted for 28.62% (33,489 out of 117,009) of all pediatric ophthalmic patients who received surgical intervention during this timeframe. Detailed subgroup characteristics for both chalazion and non-chalazion patients, along with significance testing, are presented in Table 1. The geographic distribution of the 22 hospitals is illustrated in Supplementary Material 1. The proportion of ophthalmic surgeries attributed to chalazion excision exhibited relative stability over the seven years, fluctuating between 25% and 33% (Figure 2A).

**Table 1:** Demographic characteristics.

Characteristic	Non-chalazion (n, %)	Chalazion (n, %)	Χ^2^	P
Total	83520 (71.38)	33489 (28.62)		
**Sex**			1087.68	<0.0001
Male	46837 (56.08)	15215 (45.43)		
Female	36683 (43.92)	18274 (54.57)		
**Age group**			18995.68	<0.0001
Age < 1	10494 (12.56)	202 (0.60)		
Age 1-3	22314 (26.72)	22137 (66.10)		
Age 4-6	25211 (30.19)	8646 (25.82)		
Age 7-12	23074 (27.63)	2384 (7.12)		
Age 13-18	2427 (2.91)	120 (0.36)		
**Year**			-	
2016	13884 (16.62)	4541 (13.5)		
2017	15241 (18.25)	4012 (11.98)		
2018	16421 (19.66)	4471 (13.35)		
2019	17839 (21.36)	5092 (15.20)		
2020	16455 (19.70)	5445 (16.26)		
2021	19707 (23.60)	5594 (16.70)		
2022	17462 (20.91)	4334 (12.94)		
**Seasons**			2336.428	<0.0001
Spring	18192 (21.78)	9025 (33.16)		
Summer	29317 (35.10)	6921 (19.10)		
Autumn	17815 (21.33)	8848 (33.18)		
Winter	18196 (21.79)	8695 (32.34)		
**Regions**			-	
North China	14954 (17.90)	6390 (19.10)		
Northeast China	3687 (4.41)	2411 (7.20)		
Northwest China	1318 (1.58)	5127 (15.31)		
South China	4909 (5.88)	4377 (13.07)		
East China	27027 (32.36)	8347 (24.92)		
Central China	21964 (26.30)	6820 (20.36)		
Southwest China	9661 (11.57)	17 (0.05)		
Total	83520	33489

**Figure 3:**
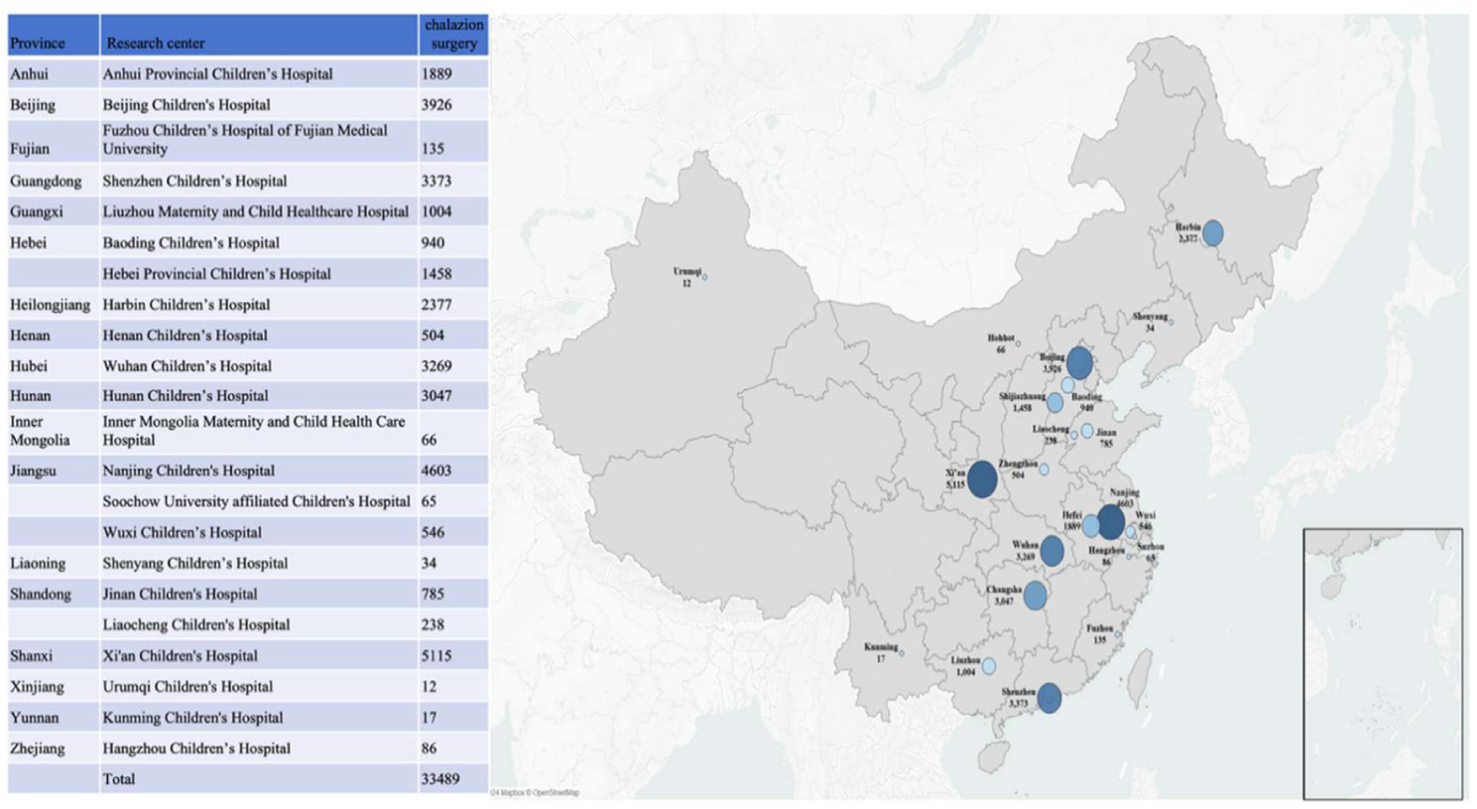
The distribution of chalazion cases under general anesthesia from 22 tertiary children’s medical centers.

GIS mapping provided a visual representation of the national distribution of cases (Figure 3). The 22 hospitals were in 22 cities across 18 provinces, encompassing various geographic regions, each characterized by varying climates, levels of development, and ethnic diversity. The cities with the highest surgical cases included Xi'an, Nanjing, Beijing, Shenzhen, and Wuhan. Compared with the proportion of non-chalazion ophthalmic surgeries performed under general anesthesia in the same regions, the proportion of chalazion surgeries among all ophthalmic surgeries under general anesthesia was significantly higher in northern China (Table 1). After excluding outliers, the proportion of chalazion surgeries among total surgeries was 29.25% (12217/41767) in northern China and 23.47% (16145/68797) in southern China. The chi-square test demonstrated a statistically significant difference between the two regions (*p* < 0.001), with a higher proportion of chalazion surgeries observed in northern China.

In the chalazion cohort, females comprised 54.57% (18,274/33,489), which is significantly greater than the male proportion of 45.43% (15,215/33,489; *p* < 0.0001). The sex ratio (males/females × 100) for chalazion cases across all age subgroups, except for infants under one year, ranged from 50 to 84, consistently lower than the ratios observed for non-chalazion ophthalmic surgical cases, which ranged from 104.5 to 127 (Figure 2B). After excluding the infant group, the overall proportion of female patients differed significantly across the remaining four age groups (χ² = 10.94, *p* = 0.012). Pairwise comparisons with Bonferroni correction revealed that only the adolescents (13 to 18 years) showed a significantly higher female proportion compared with each of the other groups (*p* = 0.006 vs 1 to 3 years, 0.007 vs 4 to 6 years); no statistically significant differences were detected among the 1 to 3, 4 to 6, and 7 to 12-year groups (all *p* > 0.008, with a significance threshold set at *p* < 0.05/6). Therefore, excluding the infant group, only the adolescents (13–18-year age) stratum showed a significantly higher proportion of female patients compared with the other age groups. The mean age of patients at the time of chalazion surgery under general anesthesia was 3.30 ± 2.05 years. Patients undergoing non-chalazion ophthalmic surgery demonstrated a significantly higher mean age of 4.85 ± 3.65 years (*p* < 0.001). Among chalazion patients within the age range of 1 to 18 years, toddlers aged 1 to 3 years represented a substantial majority, accounting for 66.10% of all chalazion excisions (Figure 2C). Furthermore, a significant decrease in chalazion surgical cases was recorded among school-aged children (ages 7 to 12 years) and adolescents (ages 13 to 18 years) (Figure 2C).

The seasonal subgroup analysis (Table 1) revealed that non-chalazion ophthalmic surgeries were predominantly performed in the summer, accounting for 35.10% (29,317 / 83,520). Conversely, chalazion surgeries showed a marked reduction during the summer when compared to other seasons, with a statistically significant difference (*p* < 0.0001). This finding suggests a significant seasonal decline in surgical cases for chalazion during the summer (Figure 2D).

In multivariable binary logistic regression, the authors examined factors associated with undergoing chalazion surgery under anesthesia, stratified by gender, age, season, and geographic region (Table 2). The assumptions for logistic regression were verifiedThese results revealed a significant gender difference: compared with male patients, female patients had a 1.52-fold higher likelihood of requiring chalazion surgery under anesthesia (aOR = 1.52, 95% CI: 1.47-1.55, *p* < 0.0001). Age was categorized into subgroups, considering a more stable baseline, with school-age children (7 to 12 years) serving as the reference group. All other age groups showed statistically significant differences in chalazion compared with the reference group (all *p*-values < 0.001). The 1-3 years group had 9.6-fold higher adjusted odds compared to the reference, greatly surpassing all other age groups (aOR = 9.60, 95% CI: 9.17-10.05, *p* < 0.001), and increased associated factor associated with undergoing chalazion surgery under general anesthesia was also seen in children aged 4-6 years (aOR = 3.32, 95% CI: 3.16-3.49, *p* < 0.001). Seasonal analysis, using summer as the reference, showed a 68% and 71% increased likelihood in autumn (aOR = 1.68, 95% CI: 1.61-1.75, *p* < 0.0001) and winter (aOR = 1.71, 95% CI: 1.64-1.77, *p* < 0.0001), respectively. Additionally, regional analysis indicated that children in northern China had an 83% higher likelihood of undergoing chalazion surgery under anesthesia than children in southern China (aOR = 1.83, 95% CI: 1.77-1.88, *p* < 0.0001).

**Table 2:** Multivariable logistic regression of associated factors for chalazion surgery under anesthesia across different clinical characteristics.

Characteristic	Adjusted Odds Ratio*	95% CI	P
**Sex (male ref)**	1.52	(1.47 to 1.55)	<0.0001
**Age group (Age 7-12 ref)**			
Age < 1	0.19	(0.16 to 0.22)	<0.001
Age 1-3	9.60	(9.17 to 10.05)	<0.001
Age 4-6	3.32	(3.16 to 3.49)	<0.001
Age 13-18	0.48	(0.40 to 0.58)	<0.001
**Seasons (summer ref)**			
Spring	1.36	(1.30 to 1.41)	<0.0001
Autumn	1.68	(1.61 to 1.75)	<0.0001
Winter	1.71	(1.64 to 1.77)	<0.0001
**Regions (South China ref)**			
North China	1.83	(1.77 to 1.88)	<0.0001

*All the factors listed in this table were included in the same multivariable logistic regression model.

## Discussion

Chalazion is generally considered a self-limiting condition that frequently resolves through conservative interventions such as warm compresses, eyelid hygiene, and topical antibiotics [1,9]. Previous studies indicated that between 15.21% - 39.4% of patients eventually require surgical intervention [10,11]. From the lower estimate of 15.21% children with chalazion needing surgery, it can be inferred that the actual population prevalence of childhood chalazion is substantially higher. In our findings, approximately 30% of ophthalmic surgeries were performed for chalazion, a consistently high frequency in seven years, which highlights its significance as a common disease in pediatric ophthalmic surgeries and the necessity for pediatric ophthalmologists to be proficient in this procedure.

Previous studies indicated that chalazion is more prevalent among preschool-aged children. In a study of 224 children, the mean age at the initial outpatient clinical diagnosis of chalazion was 3.5 ± 2.0 years (range 0.5-11.2 years) [15]. Another study reported 91 pediatric chalazion cases, with 30 cases (33%) aged 2 years and younger, and 38 cases (42%) aged 3-6 years old [16]. This seven-year, multicenter, cross-sectional study revealed that children aged 1-3 years comprised 66.10% of the total cohort undergoing general anesthesia for chalazion surgery. This age distribution aligns with the peak incidence reported by Sorensen et al., who analyzed 649 young children receiving general anesthesia for chalazion excision in tertiary hospitals (range 0.5-18.5 years) [17]. The age-specific observed is likely attributable to interactions among anatomical, meibum composition, microbial, and micronutrient [[18], [19], [20]]. Firstly, in comparison to adults, young children have fewer meibomian acini and significantly narrower ductal orifices, which predispose them to obstruction [19]. Secondly, the meibum in children exhibits a pro-obstructive lipid composition, characterized by elevated levels of cholesterol esters (CEs) and reduced wax esters (WEs) [21,22]. This hyperviscous secretion alters rheological properties that facilitate stasis and promote granulomatous inflammation. Thirdly, the immature local immune microenvironment in toddlers may allow for pathogenic colonization by Staphylococcus aureus, Staphylococcus epidermidis, and Cutibacterium acnes [3]. Additionally, *demodex* infestation, which is increasingly identified in pediatric blepharitis, is significantly associated with chalazion [5,23,24]. The lower frequency of anesthetized surgical interventions among adolescents aged 7-18 years may be due to greater glandular maturity, improved eyelid hygiene, higher rates of spontaneous resolution, and better responses to conservative and intralesional treatments within this age group; additionally, some minor procedures might be performed without anesthesia in this population.

In comparison with Western data, Evans et al. identified racial/ethnic disparities and insurance status as key drivers, with no racial difference in the need for surgical intervention. In contrast, our large-scale Chinese multicenter study highlights prominent geographic (North vs. South) and seasonal (autumn/winter) factors as associated. These differences suggest that environmental influences may play a more dominant role in the Chinese pediatric population. Our database recorded the timing of the surgery rather than the onset of chalazion; these results should not be explained as direct evidence that chalazion more frequently begins in colder seasons. Rather, they indicate that surgeries were more often performed in autumn and winter and in northern regions. Several mechanisms may reasonably contribute to this phenomenon. The significantly elevated associated factors of chalazion observed in North China and during colder seasons (autumn and winter) suggest that environmental factors, particularly ambient temperature, play an important role. Meibum typically exhibits a melting point range between 32°C and 45°C; [25] yet winter temperatures in northern China often drop well below this range. Prolonged cold exposure likely increases meibum viscosity, impeding its delivery to the ocular surface, raising the risk of ductal obstruction, and promoting chalazion formation.

In addition to climatic and seasonal factors, nutritional factors, particularly vitamin A (VA) status, may contribute to the geographic disparity in pediatric chalazion. Studies in southwest China have demonstrated that low serum VA levels are significantly associated with chalazion, especially multiple lesions [26], while oral VA supplementation markedly reduces recurrence rates in VA-deficient children [27]. National nutrition surveys have highlighted suboptimal dietary VA intake and regional disparities among Chinese children [28,29]. Compelling evidence links VA deficiency to metaplasia of glandular, ciliated, and mucus‑secreting epithelia, accompanied by hyperkeratosis of keratinizing epithelium. The resulting epithelial hyperkeratosis may obstruct meibomian gland ducts and cause accumulation of secretory material, thus predisposing individuals to chalazion [30]. These nutritional gaps, together with potential regional variations in diet and socioeconomic factors, may partially explain the higher prevalence in northern China. However, since our study did not measure environmental exposure, nutritional status, or lesion onset, these explanations should be regarded as hypotheses rather than confirmed causal mechanisms.

Another finding in our study demonstrates a consistent female predominance (54.57%) in pediatric patients except infants under 1 year old who were undergoing surgical management for chalazion, aligning with the sex disparity incidence in pediatric cohorts [11,17,31]. Especially, excluding the infant group, the proportion of female patients in the adolescent group (13 to 18 years) is significantly higher than in the other age groups. Individuals in this age group have essentially entered puberty. The influence of sex hormones cannot be overlooked. The authors hypothesize that the postulated role of androgens in meibomian gland function, combined with lower androgen levels in females, may alter meibomian gland epithelial cells, affecting lipid synthesis and secretion, thereby providing a plausible explanation for the observed sex-based disparity in pathology [32]. However, direct measurements of androgen levels in pediatric cases are lacking in this study. Future studies measuring serum hormones in patients with chalazion are warranted.

This study has several limitations. First, it was a hospital-based retrospective analysis limited to children who underwent chalazion surgery under general anesthesia at participating tertiary centers. Therefore, our findings reflect the epidemiology of surgically managed chalazion under general anesthesia rather than the population incidence, true prevalence, or full clinical spectrum of childhood chalazion. Second, only the date of surgery was recorded, while the date of chalazion onset was unavailable. As a result, there may have been a variable interval between symptom onset and surgical intervention, and the observed autumn/winter pattern should be interpreted as a seasonal pattern in the timing of surgery rather than direct evidence of the seasonality of disease onset. Third, because population denominators were unavailable, the authors could not calculate per-capita rates or adjust for regional differences in population distribution, urban-rural disparities, healthcare access, or provider availability. Accordingly, the observed North-South difference should not be interpreted as a population-based regional comparison, and residual confounding related to referral patterns, institutional case mix, and variation in surgical thresholds cannot be excluded. Fourth, important clinical variables were unavailable, including lesion size, duration, recurrence history, severity of meibomian gland dysfunction, prior treatment response, serum vitamin A levels, microbiological findings, and the exact interval from onset to surgery. The absence of these data limited our ability to assess disease severity, explore underlying mechanisms, and control for residual confounding. Future prospective, population-based studies incorporating onset dates, detailed clinical characteristics, nutritional measures, and environmental exposures are warranted to better understand the mechanisms underlying the observed associations.

Overall, this large-scale multicenter cross-sectional study identifies specific demographic and seasonal factors associated with childhood chalazion requiring surgery under anesthesia in China. Significant independent predictors of undergoing chalazion surgery under general anesthesia included age 1-3 years, being female, residence in Northern China, and surgery during autumn and winter. After excluding the infant group, the proportion of female patients undergoing general anesthesia surgery was significantly higher in the 13 to 18‑year adolescent group compared with the 1 to 3‑year and 4 to 6‑year younger children groups. The high proportion of chalazion among pediatric ophthalmic surgeries underscores its considerable clinical burden. These findings emphasize the substantial surgical impact of childhood chalazion in tertiary pediatric ophthalmic practice and support increased focus on early conservative management and eyelid hygiene in subgroups.

## Funding

This research was funded by the National Natural Science Foundation of China (No. 82371093), the Yangzhou Natural Science Foundation, Joint Special Project for Health and Wellness (No. YZ20250324).

## Declaration of generative AI in scientific writing

No generative artificial intelligence (AI) or AI-assisted technologies were used in any part of the manuscript preparation, including data analysis, figure creation, language editing, or writing.

## Data availability statement

The data used in this study were obtained from the Futang Updating Medical Records (FUTURE) system. Due to institutional and ethical restrictions related to patient privacy and data-sharing agreements, the raw data are not publicly available. De-identified data may be provided by the corresponding author upon reasonable request and with permission from the relevant data custodians.

## Ethics approval

All procedures and data collection were conducted in compliance with the tenets of the Declaration of Helsinki. This study has received ethical approval from the medical ethics committee of the Beijing Children’s Hospital, Capital Medical University(2025-202-D).

## Consent to participate

The patients’ informed consent was not required because the authors retrospectively collected data.

## Consent for publication

All authors have read and agreed to the published version of the manuscript. And neither this manuscript nor one with substantially similar content under our authorship has been published or is being considered for publication elsewhere.

## Authors contribution statement

Jingjing Jiang and Li Li contributed to the design and revision of the manuscript, while Xinyu Wang, Guoshuang Feng, and Chunni Zhang played key roles in the data analysis. Chang Liu and Jingjing Jiang were responsible for the initial drafting of the manuscript. All authors have reviewed and approved the final version of the published paper.

## Conflicts of interest

The authors declare no conflict of interest. The funders had no role in the design of the study, the collection, analysis, or interpretation of data, the writing of the manuscript, or the decision to publish the results.

## Appendix Supplementary materials

Image, application 1
