# Supplementary material for: Epidemiology and associated factors of childhood chalazion in China: a seven-year, hospital-based, multicenter, cross-sectional study
Source: J Pediatr (Rio J). 2026 May 29;102(4):101556. doi: 10.1016/j.jped.2026.101556 (PMC13241733; doi:10.1016/j.jped.2026.101556)
Supplement: Supplementary file 1 [file mmc1.docx]

**JPED-D-25-00574_Supplementary Material**


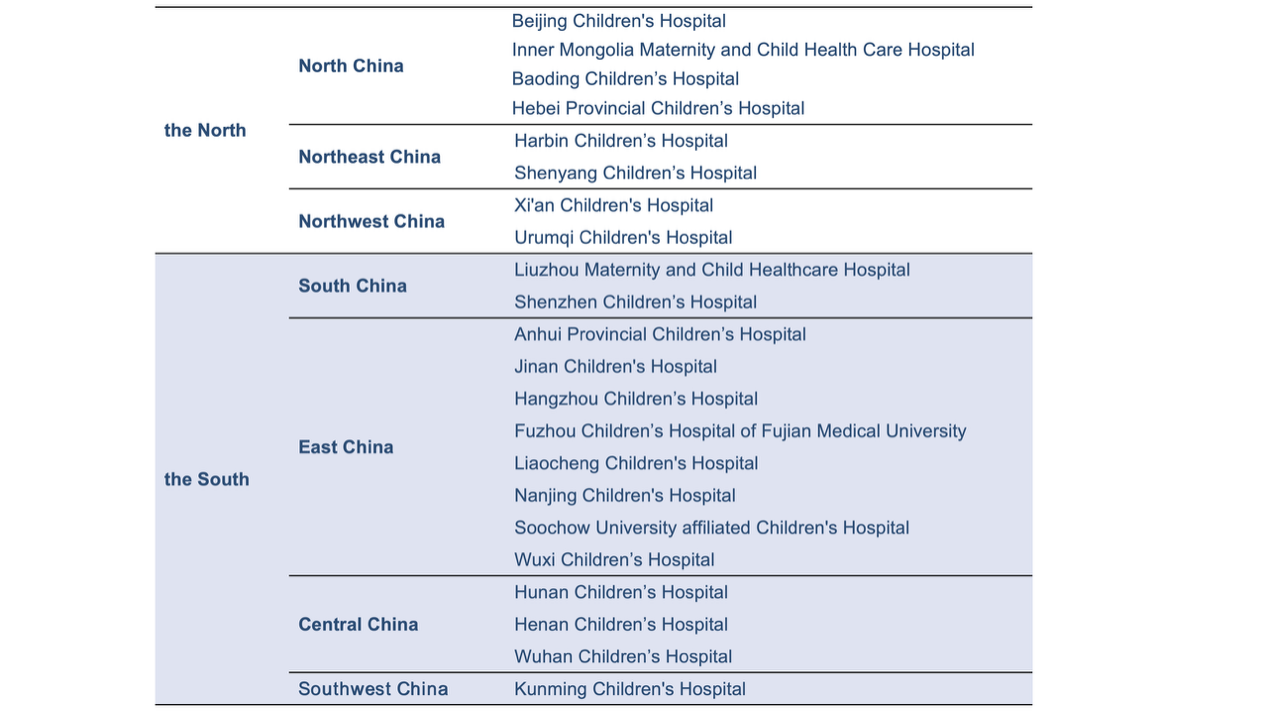


**Supplementary Materials 1** The regional distribution of the 22 tertiary children’s medical centers.
